# Regional Intestinal Drug Permeability and Effects of Permeation Enhancers in Rat

**DOI:** 10.3390/pharmaceutics12030242

**Published:** 2020-03-08

**Authors:** David Dahlgren, Maria-Jose Cano-Cebrián, Tobias Olander, Mikael Hedeland, Markus Sjöblom, Hans Lennernäs

**Affiliations:** 1 Department of Pharmacy, Division of Biopharmaceutics, Uppsala University, 752 36 Uppsala, Sweden; david.dahlgren@farmaci.uu.se (D.D.); olander92@hotmail.com (T.O.); 2 Department of Pharmacy and Pharmaceutical Technology and Parasitology, University of Valencia, 46010 València, Spain; Maria.Jose.Cano@uv.es; 3 Department of Medicinal Chemistry, Analytical Pharmaceutical Chemistry, Uppsala University, 752 36 Uppsala, Sweden; mikael.hedeland@ilk.uu.se; 4 Department of Chemistry, Environment and Feed Hygiene, National Veterinary Institute (SVA), 751 89 Uppsala, Sweden; 5 Department of Neuroscience, Division of Physiology, Uppsala University, 752 36 Uppsala, Sweden; Markus.Sjoblom@neuro.uu.se

**Keywords:** regional intestinal permeability, permeation enhancers, absorption-modifying excipients, oral peptide delivery, intestinal perfusion, pharmaceutical development

## Abstract

Sufficient colonic absorption is necessary for all systemically acting drugs in dosage forms that release the drug in the large intestine. Preclinically, colonic absorption is often investigated using the rat single-pass intestinal perfusion model. This model can determine intestinal permeability based on luminal drug disappearance, as well as the effect of permeation enhancers on drug permeability. However, it is uncertain how accurate the rat single-pass intestinal perfusion model predicts regional intestinal permeability and absorption in human. There is also a shortage of systematic in vivo investigations of the direct effect of permeation enhancers in the small and large intestine. In this rat single-pass intestinal perfusion study, the jejunal and colonic permeability of two low permeability drugs (atenolol and enalaprilat) and two high-permeability ones (ketoprofen and metoprolol) was determined based on plasma appearance. These values were compared to already available corresponding human data from a study conducted in our lab. The colonic effect of four permeation enhancers—sodium dodecyl sulfate, chitosan, ethylenediaminetetraacetic acid (EDTA), and caprate—on drug permeability and transport of chromium EDTA (an established clinical marker for intestinal barrier integrity) was determined. There was no difference in jejunal and colonic permeability determined from plasma appearance data of any of the four model drugs. This questions the validity of the rat single-pass intestinal perfusion model for predicting human regional intestinal permeability. It was also shown that the effect of permeation enhancers on drug permeability in the colon was similar to previously reported data from the rat jejunum, whereas the transport of chromium EDTA was significantly higher (*p* < 0.05) in the colon than in jejunum. Therefore, the use of permeation enhancers for increasing colonic drug permeability has greater risks than potential medical rewards, as indicated by the higher permeation of chromium EDTA compared to the drugs.

## 1. Introduction

The rat single-pass intestinal perfusion (SPIP) model investigates epithelial membrane permeability, a key biopharmaceutical variable in drug absorption following oral intake [1]. The model is therefore frequently used in pharmaceutical development to evaluate the potential success of a drug, for instance with oral modified-release (MR) dosage forms. In MR dosage forms, the drug is released throughout the gastrointestinal (GI) tract prior to absorption so the regional intestinal permeability needs to be sufficiently high in both the small and large intestine. The rat and human small intestine have similar drug intestinal absorption profiles and transporter expression patterns, but differ in their enzymatic metabolism [2]. Differences in absorption from the rat and human colon have not been extensively compared, but a recent meta-analysis of rat SPIP data reports regional differences in drug permeability for 42 drugs in this species [3]. 

How relevant for humans are the regional intestinal drug permeability values determined in the rat SPIP model? It is difficult to answer this because of the limited amount of human reference permeability data from the lower GI tract (colon), and inter-laboratory variability in permeability determinations using the rat SPIP model [3,4]. Foremost, it is inherently difficult to accurately determine the luminal disappearance of medium-to-low permeability drugs in the SPIP model. Permeability is often overestimated for drugs with a low permeability because differences in the perfusate concentrations entering and leaving the perfused segment may be too small for accurate quantification. To circumvent this problem for low-permeation compounds, the drug permeability can be determined on the basis of plasma appearance data of intact drug (corrected for first-pass extraction) [5]. For instance, a recent study in the rat jejunum showed that the permeability value of the low permeability drugs atenolol and enalaprilat was >10 times higher in the same rat when determined from luminal disappearance, compared to plasma appearance [5]. In the same rat study, as well as in a human study, there were no differences for the high-permeability compounds metoprolol and ketoprofen. Thus, the choice of determination method seems to be important only for low-permeation compounds [4]. Accordingly, there is need for an evaluation of the human in vivo predictive relevance of regional intestinal drug permeability values determined from plasma appearance in the rat SPIP model.

The rat SPIP model may also be used to investigate regional intestinal differences in how pharmaceutical excipient(s) affect drug permeation and overall absorption rate. This is especially relevant because of the renewed interest in permeation enhancers (PE), also called absorption-modifying excipients (AME), for enabling oral administration of low-permeation compounds, for example, peptides [6,7]. Some advocates of this formulation approach propose the colon as a potential target for PEs, because the colon has a longer residence time, its mucosa may be more easily affected, and it does not have the higher peptidase activity of the upper GI tract [8,9]. 

However, few comparisons of the small and large intestine in rat have been made on the direct permeability effects of PEs in the same laboratory. Even fewer comparisons have used in vivo models, which are substantially more resilient to intestinal PE effects than in vitro models such as cell- and tissue-based systems [10]. Accordingly, there is a need for a systematic evaluation of PE effects in the small and large intestine in the more relevant in vivo permeability models, such as SPIP.

The primary objective of this rat SPIP study was to investigate the regional intestinal differences (jejunum vs colon) in lumen-to-blood drug permeability, as determined from plasma appearance data. Permeability values were determined at both pH 6.5 and 7.4 for two low permeability model drugs (atenolol and enalaprilat) and for two high-permeation ones (ketoprofen and metoprolol). The second objective was to evaluate the relevance of the rat model, by comparing the regional intestinal permeability values with reported values of three model drugs (not enalaprilat) in human, as determined from plasma drug appearance. The third objective was to investigate the effect in the rat colon of four PEs with different mechanisms of action: sodium dodecyl sulfate (SDS), chitosan, ethylenediaminetetraacetic acid (EDTA), and sodium caprate. These four PEs have previous rat jejunal reference values at the same luminal concentrations determined at our laboratory [11,12]. The PE effects were evaluated based on changes in permeability of the four model drugs, and in blood-to-lumen clearance of ^51^chromium-labeled ethylenediaminetetraacetate (CL_Cr-EDTA_), an established clinical marker for mucosal barrier integrity [13].

## 2. Materials and Methods

### 2.1. Active Pharmaceutical Ingredients, Pharmaceutical Excipients and Other Chemicals

Four model compounds were selected: atenolol, enalaprilat, ketoprofen, and metoprolol. Biopharmaceutical classification (BCS) and some physicochemical properties for the four drugs are summarized in Table 1. Four PEs with different mechanisms of action were selected: SDS (anionic surfactant), sodium caprate (fatty acid), chitosan (polysaccharide), and EDTA (chelating agent). Atenolol and metoprolol tartrate were provided by AstraZeneca AB (Mölndal, Sweden). Enalaprilat, ketoprofen, sodium caprate, SDS, bovine albumin (A2153), EDTA, and inactin (thiobutabarbital) were purchased from Sigma-Aldrich (St. Louis, MO, USA). Sodium phosphate dibasic dihydrate (Na_2_HPO_4_·2H_2_O), potassium dihydrogen phosphate (KH_2_PO_4_), sodium hydroxide (NaOH), and sodium chloride (NaCl) were purchased from Merck KGaA (Darmstadt, Germany). ^51^Cr-EDTA was purchased from PerkinElmer Life Sciences (Boston, MA, USA). Chitosan hydrochloride (molecular mass 40-300 kDa, degree of acetylation 8.8%) was purchased from Kraeber and Co GmbH (Ellerbek, Germany). Parecoxib (dynastat) was obtained from Apoteket AB, Uppsala, Sweden. 

### 2.2. Study Formulations

Eight isotonic (290 mOsm) phosphate-buffered perfusates were prepared, each containing all four drugs atenolol, enalaprilat, ketoprofen, and metoprolol at 100 µM. There were two control solutions at pH 6.5 and 7.4 containing no PEs, and six test formulations containing PEs. The phosphate buffer strength was 8 mM at pH 6.5, and 80 mM at pH 7.4 to avoid a reduction in pH during the perfusion. Five of the test formulations were perfused at pH 6.5 and contained one of the following PEs in solution: SDS at 1 and 5 mg/mL (3.5 and 17.3 mM), EDTA at 1 and 5 mg/mL (3.4 and 17.1 mM), and chitosan at 5 mg/mL (≈30 µM). One of test formulations was perfused at pH 7.4 and contained a suspension of sodium caprate at 10 mg/mL (51 mM). The higher pH in the perfusate was used for caprate as it has no permeation enhancing effect at pH 6.5 in either the rat or human SPIP models, as its solubility is higher at pH 7.4 (2 vs. 5 mg/mL) [11,15]. The PE concentrations of 1, 5, and 10 mg/mL correspond to oral doses of 0.2, 1.0, and 2.0 g administered with 200 mL water, as these values are previously shown to affect the intestinal permeability of low-permeation model compounds in the rat SPIP model [11,12].

The preparation procedure of the perfusion formulations (100 mL) is described in detail earlier [12]. No incompatibility, degradation, or apparent binding to glass/plastic of the study compounds in solution (pH 6.5, 37 °C) was observed during 4 h. Osmolarity was determined (after addition of all perfusate constituents, e.g., salt, PE, water) by freezing-point depression using a Micro Osmometer (Model 3MO; Advanced Instruments, Needham Heights, MA, USA). 

### 2.3. Animals and Study Design

The surgical procedure and experimental setup of the rat SPIP experiment has been previously described [12]. The study was approved by the local ethics committee for animal research (no: C64/16) in Uppsala, Sweden. In short, male Han Wistar rats (strain 273) from Charles River Co. (Cologne, Germany), weight 270–420 g, were used. On the study day, the rats were anesthetized using an intraperitoneal injection of a 5% *w*/*v* inactin solution (180 mg/kg). Body temperature was maintained at 37.5 ± 0.5 °C. Systemic arterial blood pressure was continuously recorded to validate the condition of the animal. This was done by connecting an arterial catheter to a transducer operating a PowerLab system (AD Instruments, Hastings, UK).

At the SPIP experiment, the abdomen was opened along the midline and a jejunal (10–12 cm) or colonic (6–12 cm) segment was cannulated, covered with polyethylene wrap, and placed outside the abdomen [5]. The bile duct was cannulated to avoid pancreaticobiliary secretion into the duodenum at the jejunal perfusion. After completion of surgery, ^51^Cr-EDTA was administered intravenously (iv) as a bolus of 75 µCi (0.4 mL), followed by a continuous iv infusion at a rate of 50 µCi per hour (1 mL/h) for the duration of the experiment. During the first 30 min following surgery, each small and large intestinal segment was single-passed perfused with 37 °C, phosphate-buffered saline (6 mM) at pH 6.5 or 7.4. This stabilized cardiovascular, respiratory, and intestinal functions and the ^51^Cr-EDTA levels in the blood (plasma). The length and wet tissue weight of each intestinal segment was determined after the experiment. The single-pass perfusion rate was at all times 0.2 mL/min (peristaltic pump, Gilson Minipuls 3, Le Bel, France).

Each of the six PE experiments was performed in the colon and was divided into two parts. In the first part, the segment was perfused with the control buffer solution (containing model compounds but no PE) for 60 min. In the second part, the segment was perfused for 75 min with one of the six test formulations, containing model compounds and one of the following PEs: SDS at 1 or 5 mg/mL, EDTA at 1 or 5 mg/mL, chitosan at 5 mg/mL (pH 6.5), and caprate at 10 mg/mL (pH 7.4). The six PE experiments were designed so that each rat was its own control. For regional intestinal comparisons, all the above PE concentrations and pH values were previously evaluated in the jejunum, at our laboratory and using the same experimental design. 

To evaluate regional intestinal differences in basal permeability values of the four model drugs, two perfusions were also performed in the jejunum using only the control solutions (no PE) for 60 min, at pH 6.5 and 7.4. This established a basal permeability value for comparison with the values determined in the control period of the PE experiments in the colon.

All experimental periods started with a rapid filling (<30 s) of the whole segment with the perfusate (about 1.5 mL for a 10-cm segment). The intestinal segment and perfusates were kept at 37 °C and all outgoing perfusate was quantitatively collected and weighed at 15-min intervals. 

Blood samples of <0.3 mL were collected from the femoral artery for a maximum volume of 4 mL during each experiment. All sampled blood volumes were replaced by an equivalent volume of saline (0.9% NaCl) solution with 70 mg/mL bovine serum albumin. Blood was sampled at 15-min intervals for 135 min (9 samples) in each of the six PE experiments, and for 60 min (4 samples) in the jejunal controls. The blood samples were put on ice and centrifuged (5000× *g*, 3 min at 4 °C) within 10 min. 100 μL of the plasma was transferred to 500 µL microtubes and stored at −20 °C until analysis. 

### 2.4. Determination of Blood-to-Lumen Jejunal ^51^Cr-EDTA Clearance (CL_Cr-EDTA_)

In the six PE experiments, all luminal perfusates and blood plasma were analyzed at 0 and 135 min for ^51^Cr activity (cpm) in a gamma counter (1282 Compugamma CS, Pharmacia AB, Uppsala, Sweden). A linear regression analysis of the plasma samples was made to obtain a corresponding plasma value for each perfusate sample. The blood-to-lumen CL_Cr-EDTA_ was calculated using Equation (1) [16].

(1) $$CL_{Cr-EDTA}=\frac{C_{perfusate\;}\times \;Q_{in}}{C_{plasma\;}\times \;tissue\;weight\;}\times 100$$

where C_perfusate_ and C_plasma_ is the activity in the perfusate and plasma (cpm/mL), and Q_in_ is the flow rate (mL/min). CL_Cr-EDTA_ was determined during the last 45 min for the control solution and during the last 60 min for the test solutions, of which the first 15 min of each period were for equilibration. The mean CL_Cr-EDTA_ value of the two perfusion periods was regarded as representative for each individual rat.

### 2.5. Bioanalysis

The plasma concentrations of atenolol, metoprolol, enalaprilat and ketoprofen were determined using Ultra-High Performance Liquid Chromatography coupled to Tandem Mass Spectrometry. The method used has been previously published [17]. The only modification was that the lower limit of quantification for ketoprofen was decreased to 52 nM in this study. 

### 2.6. Intestinal Effective Permeability (P_eff_) Calculation

Jejunal and colonic lumen-to-blood effective permeability (P_eff_) of the four model compounds was determined based on a modification of the method described by Sjögren et al., 2015 [18]. This method has been successfully implemented in human, dog and rat [4,18,19,20,21]. In short, an input rate was acquired by deconvolution of the plasma concentration–time profiles following the intestinal perfusion using Phoenix software version 8.2 (Certara USA, Princeton, NJ, USA). Previous intravenous pharmacokinetic data from a two-compartment analysis of the model drugs in Han Wistar rats was used as impulse response in the deconvolution [12]. An absorption rate was then calculated by compensating for first-pass extraction (F_firstpass_) of each compound in the rat intestine and liver. The F_firstpass_ values for atenolol (1.0), enalaprilat (0.99), ketoprofen (0.99), and metoprolol (0.22) were based on literature data for the fraction of the model compound as follows: (i) the amount metabolized/excreted in the rat liver; (ii) plasma CL values derived from the two-compartment analysis of the intravenous plasma data; and (iii) an assumed rat-liver blood flow of 47 mL/min/kg [22,23,24]. The P_eff_ (cm/s) was then calculated by relating the absorption rate to the intestinal luminal area using Equation (2):

(2) $$P_{eff}=\frac{absorption\;rate}{A\;\times \;C}$$

where *A* is the area of the exposed intestinal segment described as a smooth cylinder with a radius of 0.2 cm, and C is the concentration entering the luminal segment. 

In the six colonic PE experiments, P_eff_ was evaluated from 0 to 135 min and the mean P_eff_ value of the two perfusion periods (60-min control and 75-min test) was regarded as representative for each individual rat. In the control experiments performed in jejunum, P_eff_ was evaluated from 0 to 60 min and the mean P_eff_ value was regarded as representative for each individual rat.

### 2.7. Statistical Analysis

The sample size in each study group was six rats, on the basis of power analysis and previous perfusion studies [12,25]. Plasma concentration, P_eff_, and CL_Cr-EDTA_ values are expressed as mean ± standard deviation (SD) or standard error of the mean (SEM). The jejunal vs colonic P_eff_ ratio, is presented as well as the P_eff_ and CL_Cr-EDTA_ ratio between the 45-min control and 60-min test period in the six colonic PE perfusions (Equation (3)).

(3) $$Ratio\;\left(CL_{Cr-EDTA}\;or\;P_{eff}\right)=\frac{mean\;value\;\left(jejunum\;or\;test\;period\right)\;}{mean\;value\;\left(colon\;or\;control\;period\right)}$$

The ratio was compared using the paired student’s t-test with the Benjamini–Hochberg multiple t-test correction. Multiple comparisons between groups were performed using a two-way ANOVA with a post-hoc Holm–Sidak multiple comparison test. Log transformation of values was performed when the original measured data were heteroscedastic and not normally distributed; this was investigated using the Bartlett test. Differences were considered to be statistically significant for *p*-values < than 0.05. 

## 3. Results

### 3.1. Plasma Profiles

**Figure 1:**
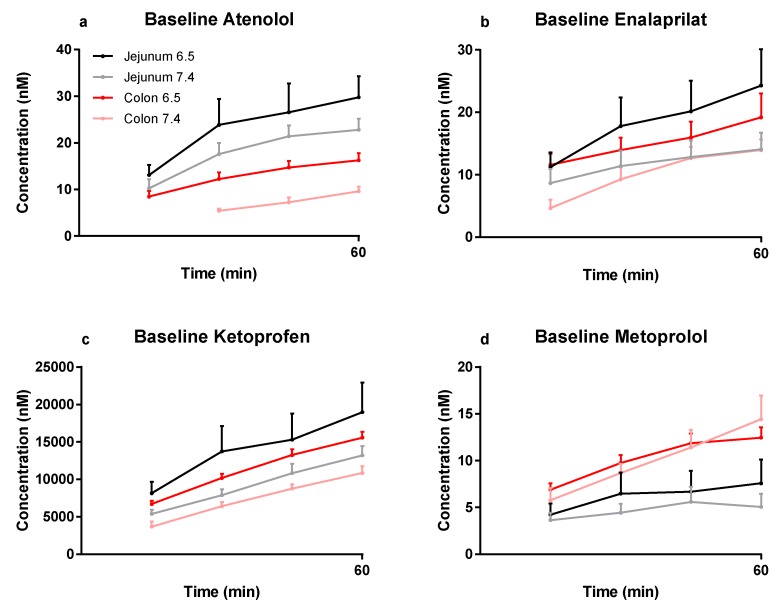
The mean ±SEM rat plasma concentration–time profiles (*n* = 30 for colon at pH 7.4, and *n* = 6 for the other three groups) of: (**a**) atenolol, (**b**) enalaprilat, (**c**) ketoprofen, and (**d**) metoprolol following single-pass jejunal and colonic perfusions of the pH 6.5 and 7.4 control solutions (0–60 min). These plasma data were used to determine regional intestinal basal P_eff_ values using Equation (2) (Table 2).

The mean (±SEM) plasma concentration–time profiles are presented in Figure 1a–d for atenolol, enalaprilat, ketoprofen, and metoprolol following the jejunal and colonic perfusions (first 60 min) of the control solutions at pH 6.5 and 7.4. These plasma concentration–time data for the selected model drugs were used to determine regional intestinal basal P_eff_ values using Equation (2). 

**Figure 2:**
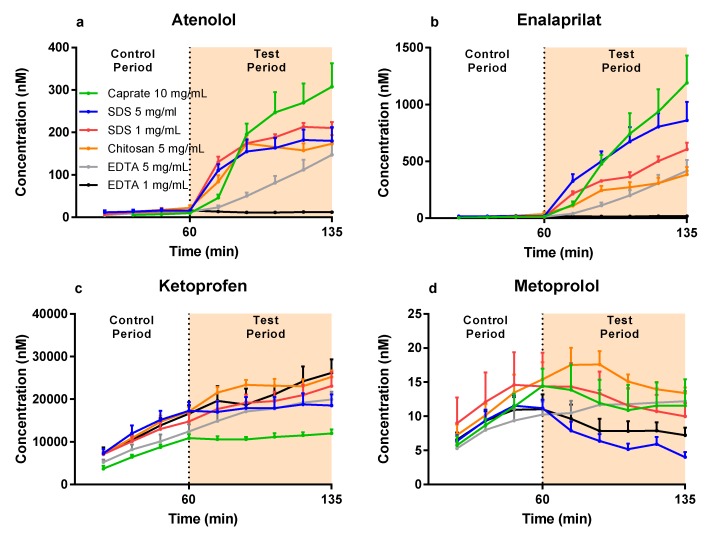
The mean ±SEM rat colonic plasma concentration–time profiles (*n* = 6) of: (**a**) atenolol, (**b**) enalaprilat, (**c**) ketoprofen, and (**d**) metoprolol following single-pass intestinal perfusions of a control solution for 60 min, followed by a 75-min perfusion of any of six test formulations containing a permeation enhancer (PE). The control solution and all test formulations contained 100 µM atenolol, enalaprilat, ketoprofen, and metoprolol. The control and test formulation perfusate pH was 6.5 for the PEs: sodium dodecyl sulfate (SDS) at 1 and 5 mg/mL, chitosan at 5 mg/mL, and ethylenediaminetetraacetic acid (EDTA) at 1 and 5 mg/mL. The control and test formulation perfusate pH was 7.4 for caprate at 10 mg/mL. All formulations were solutions, except caprate which was a suspension (its solubility at pH 7.4 is 5 mg/mL).

The mean (±SEM) plasma concentration–time profiles are presented in Figure 2a–d for atenolol, enalaprilat, ketoprofen, and metoprolol after: (i) the colonic perfusions of the control solutions (0–60 min), and (ii) then followed by the six PE-containing test formulations (60–135 min). These plasma concentration–time data were used to determine the PE-induced increase in P_eff_ ratio (test/control period) using Equation (3). 

### 3.2. Lumen-to-Blood Effective Permeability (P_eff_) of Model Drugs 

The mean (±SEM) basal jejunal and colonic P_eff_ at pH 6.5 and pH 7.4 are presented in Table 2 for atenolol, enalaprilat, ketoprofen, and metoprolol. There were no statistical (*p* < 0.05) differences in basal permeability for any of the model drugs at either pH or in any intestinal segment. 

**Figure 3:**
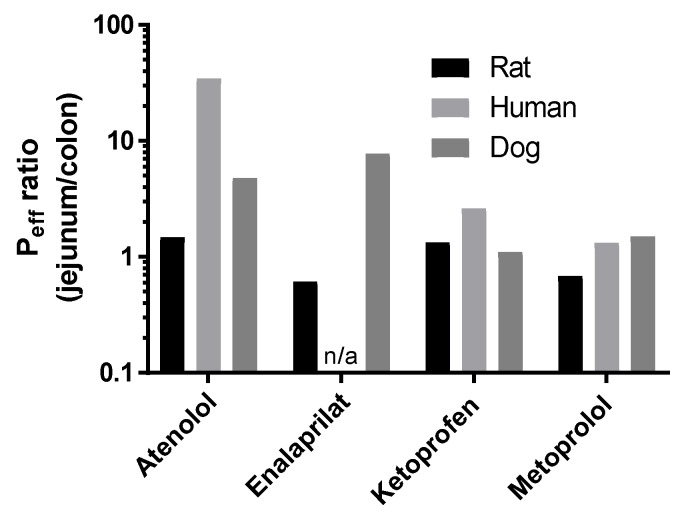
The P_eff_ ratio between the jejunum and colon at pH 6.5 in rat of atenolol, enalaprilat, ketoprofen, and metoprolol (Table 2). The historical human and dog P_eff_ ratios between the jejunum and colon at pH 6.5 of atenolol, enalaprilat (not human), ketoprofen, and metoprolol are also presented for species comparison [4,19].

The mean P_eff_ ratio between the jejunum and colon of atenolol (1.5), enalaprilat (0.6), ketoprofen (1.3), and metoprolol (0.7) at pH 6.5 are presented in Figure 3. For species comparison, Figure 3 also contains the previously published human/dog P_eff_ ratio between the jejunum and colon for atenolol (35/5), enalaprilat (not available/8), ketoprofen (2.6/1.0), and metoprolol (1.3/1.5) at pH 6.5 (plasma appearance data) [4,19].

**Figure 4:**
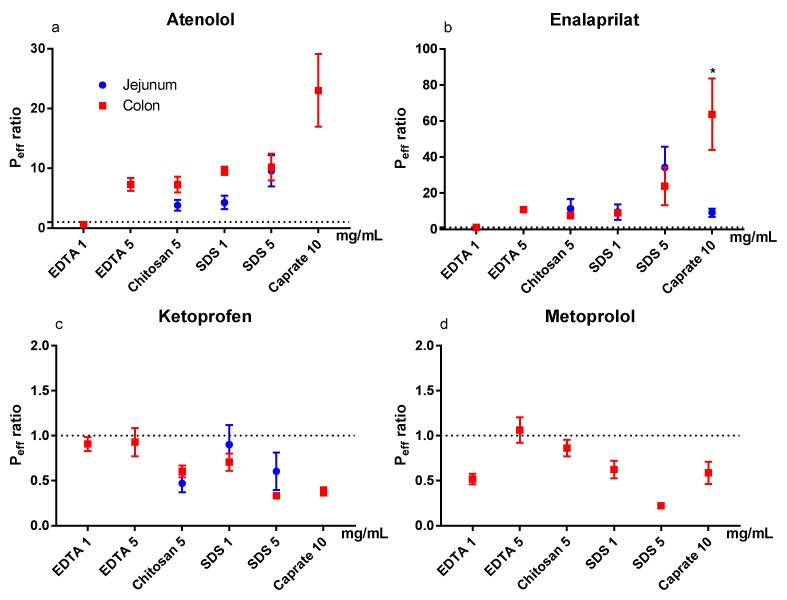
The mean ±SEM rat jejunal (historical data) and colonic lumen-to-blood intestinal effective permeability (P_eff_) ratio (*n* = 6) of: (**a**) atenolol, (**b**) enalaprilat, (**c**) ketoprofen, and (**d**) metoprolol, after intestinal perfusions of a control solution for 60 min, followed by a 75-min perfusion of any of six permeation enhancing (PE) test formulations [11,12]. The control and test formulation perfusate pH was 6.5 for the PEs: sodium dodecyl sulfate (SDS) at 1 and 5 mg/mL, chitosan at 5 mg/mL, and ethylenediaminetetraacetic acid (EDTA) at 1 and 5 mg/mL. The control and test formulation perfusate pH was 7.4 for caprate at 10 mg/mL. All formulations were solutions, except caprate which was a suspension (its solubility at pH 7.4 is 5 mg/mL). There is no jejunal historical data for metoprolol and only jejunal historical data for EDTA and caprate for enalaprilat. A * represents a significant difference in jejunal and colonic P_eff_ (two-way ANOVA, Holm–Sidak).

The mean (±SEM) P_eff_ ratio of the test and control periods for the six test formulations in the colon are shown in Figure 4a–d for atenolol, enalaprilat, ketoprofen, and metoprolol. Figure 4a–d (blue symbols) also contains previous jejunal P_eff_ ratio data of atenolol, enalaprilat, and ketoprofen for chitosan at 5 mg/mL, and for SDS at 1 and 5 mg/mL (and for enalaprilat with caprate at 10 mg/mL) [11,12]. The colon seems to be more sensitive than the jejunum to caprate at 10 mg/mL, as the P_eff_ ratio of enalaprilat was significantly (*p* < 0.05) higher in the colon. There were no statistical differences between intestinal segments for any of the other model drugs and PEs.

### 3.3. Blood-to-Lumen CL_Cr-EDTA_ Ratio

**Figure 5:**
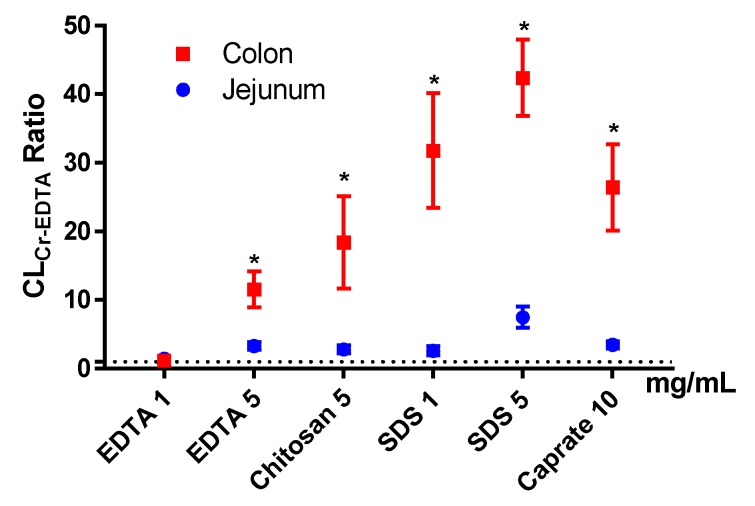
The mean ±SEM rat jejunal (historical data) and colonic blood-to-lumen ^51^Cr-EDTA clearance (CL_Cr-EDTA_) ratio (*n* = 6), after intestinal perfusions of a control solution for 60 min, followed by a 75-min perfusion of any of six permeation enhancing (PE) test formulations. The control and test formulation perfusate pH was 6.5 for the PEs: sodium dodecyl sulfate (SDS) at 1 and 5 mg/mL, chitosan at 5 mg/mL, and ethylenediaminetetraacetic acid (EDTA) at 1 and 5 mg/mL. The control and test formulation perfusate pH was 7.4 for caprate at 10 mg/mL. All formulations were solutions, except caprate which was a suspension (its solubility at pH 7.4 is 5 mg/mL). A * represents a significant difference in jejunal and colonic CL_Cr-EDTA_ ratio (two-way ANOVA, Holm–Sidak).

The mean (±SD) colonic CL_Cr-EDTA_ for the control solutions (*n* = 38) was 0.038 ± 0.050 mL/min/100 g. The mean (±SEM) CL_Cr-EDTA_ ratios between the control and test period for the six test formulations in the colon (and for previously reported jejunal data, blue symbols) are shown in Figure 5. Unlike the P_eff_ ratios, there was a significant PE-induced increase in CL_Cr-EDTA_ ratio in the colon compared to the control for all test formulations, except EDTA at 1 mg/mL. The increases were also significantly higher in the colon than in the jejunum for all test formulations.

## 4. Discussion

This rat single-pass intestinal perfusion (SPIP) study is part of a sequence of mechanistic studies to evaluate regional intestinal differences in drug absorption in different species and models. The study also evaluates the in vivo effect of permeation enhancers (PEs) on intestinal transport of model drugs/peptides and marker compounds [12,25,26,27]. The primary objective was to investigate the regional intestinal differences in lumen-to-blood effective drug permeability (P_eff_)—as determined from plasma appearance data in the rat SPIP model—and to compare it to corresponding historical human data [4]. P_eff_ was determined for two low-permeation model drugs, atenolol and enalaprilat, and for two high-permeation drugs, ketoprofen and metoprolol. 

The secondary objective was to evaluate the effect of PEs on drug permeability in the rat colon, compared to previous jejunal data. The effects were evaluated based on model drug P_eff_ and blood-to-lumen clearance of ^51^Cr-EDTA (CL_Cr-EDTA_), an established clinical marker for mucosal barrier integrity. 

A modified-release (MR) dosage form can be used to optimize plasma pharmacokinetics, dosage regimens, and improve clinical performance. MRs enable once-per-day drug administration, reduce side effects, and increase patient compliance [28]. Successful development of such a dosage form requires that the drug be absorbed in all parts of the intestines, as drug release needs to be substantially longer than the typical human small intestinal transit time of 3–5 h [29]. Reliable preclinical data on regional intestinal permeability is therefore needed early in the development of any novel MR dosage form. The rat SPIP model is commonly used to determine regional permeability data on the basis of luminal drug disappearance. However, a recent meta-analysis shows wide variability in regional intestinal permeability data between studies and between laboratories. This raises the question how relevant individual studies on rats are for in vivo predictions in humans [3]. The lack of a correlation may be related to the method of drug permeability determination. A recent rat SPIP study demonstrated that the permeability values of the low permeability drugs, atenolol and enalaprilat, is 9 to 59 times higher when determined on the basis of luminal disappearance compared to plasma appearance [5]. Therefore, the primary aim of this study was to evaluate the suitability of the rat SPIP model for measurements of human regional intestinal drug permeability on the basis of plasma appearance data [4,30].

In our study, there were only small differences in jejunal and colonic rat P_eff_ at pH 6.5 for the high-permeability compounds, ketoprofen and metoprolol, when determined from plasma appearance. This is in good agreement with regional intestinal permeability data based on luminal disappearance in the rat SPIP model, as well as with human regional intestinal permeability data based on plasma appearance [4,20,31]. Our results show that the rat SPIP model accurately predicted regional differences (jejunum vs colon) in the permeability of high-permeability drugs, regardless whether these were determined by luminal disappearance or plasma appearance.

For the low permeability drugs, atenolol and enalaprilat, plasma appearance data showed no differences in jejunal and colonic P_eff._ In contrast, the jejunal permeability of atenolol in human was 35 times higher than in colon. There is no reference value in human for enalaprilat, but in dog the corresponding jejunal value is eight-fold higher than the colonic permeability based on plasma appearance data [19]. Since the dog is well-known to have a colon that is much more permeable to drugs than that of human, presumably any reference value in human would result in a jejunal vs colonic ratio at least within the same order of magnitude as observed in dog [32]. Consequently, the plasma appearance of these two drugs suggests that the rat SPIP model is unable to accurately predict regional intestinal permeability of medium-to-low permeability drugs in human, which is also reported by others [33]. However, it should be emphasized that the rat SPIP model is still useful for evaluating a range of other biopharmaceutical, physiological, and biochemical processes. For instance, the rat jejunum is representative of human jejunal P_eff_ values determined from plasma appearance [4,5]. Therefore, the permeability data from the SPIP model will be useful for boundary BCS classification of permeability and for investigation of the potential effect of different concentrations of pharmaceutical excipients on local intestinal permeability [34].

There was a trend for a slightly higher (1.1- to 2.3-fold) jejunal and colonic permeability of all four model drugs at pH 6.5 compared to 7.4. On the basis of the pH-partitioning hypothesis, this was expected for the acid, ketoprofen, but not for the bases, atenolol and metoprolol [35]. These conflicting results indicate that parameters other than molecular charge dominate. For instance, passive membrane transport is also affected by paracellular pore selectivity, molecular elongation, and intramolecular hydrogen bonding, which might be better understood using complex molecular dynamic simulations [36]. Consequently, any pH-dependent permeability values determined in the rat SPIP model should be interpreted with care, and a linear pH-permeability relationship should not be used to predict intestinal drug transport and absorption. 

Peptide drugs with a very low intestinal stability and/or permeability are, with a few exceptions, not administered orally because of their low intestinal absorption. Their low stability can be related to stomach pH denaturation, the high concentrations of luminal gastric and pancreatic peptidases and proteinases, and the high peptidase activity in the brush border membrane of the enterocytes [7]. These issues can be partly circumvented by the formulation approaches. For instance, (1) enteric coating can prevent gastric chemical instability and peptide degradation; the (2) proteinase/peptidase inhibitors in the formulation can increase the local luminal stability of the drug; and (3) drug release may be targeted to the colon where peptidase activity tends to be lower than in the small intestine [9,37,38]. 

The low intestinal permeability of most peptides is related to their large size, low lipophilicity, and extensive hydrogen binding, all of which are physicochemical properties that predict low passive membrane transport [39]. A strategy to circumvent low intestinal permeability was recently approved for the first time in an oral product, for which a PE increased the intestinal membrane transport of semaglutide, a pharmaceutical peptide containing 31 amino acids, even though the bioavailability in dog is as low as 0.29% of the oral dose (data from patent: wo2012080471) [6]. The use of PEs has also been proposed in the colon, as the low luminal volumes and long transit time allow for high local mucosal PE concentrations at extended exposure times. Together these increase the likelihood of a positive effect on peptide membrane permeation. Accordingly, rat luminal instillation studies report a generally higher effect of PE in the colon than the jejunum on the plasma exposure of various molecular probes and peptides [8,40,41,42,43,44]. However, the rat luminal instillation model does not differentiate between PE effects on membrane permeability, and on transit/motility, dilution, and hydrodynamics in the luminal segment. This is in contrast to the SPIP model in which luminal and experimental conditions are controlled [10]. Therefore, our study performed a systematic evaluation of the effect of four permeation enhancers with different mechanisms of action at different luminal concentrations in colon. 

In our rat SPIP study, all PEs (except EDTA at 1 mg/mL) increased the P_eff_ of the two low permeability drugs, atenolol and enalaprilat. However, for the vast majority of the PEs in this study, the increase in P_eff_ ratio was not different from what we have previously observed in the jejunum in our laboratory [11,12]. This is in stark contrast to the significantly higher effect of all PEs in this study on CL_Cr-EDTA_ ratio in the colon compared to the jejunum. The substantially higher effect on the transport of the clinical marker for mucosal integrity and damage, compared to drug absorption, indicates a greater risk for tissue damage than medical benefit in using PE for increasing colonic drug absorption. On the basis of the data from our study, we agree with other reports that the rationale is weak for colonic targeting of systemically acting drugs/peptides in combination with PEs [45]. 

In conclusion, this rat SPIP study showed no difference in jejunal and colonic permeability determined from plasma appearance data of two low permeability model compounds (atenolol and enalaprilat) and two high-permeability ones (ketoprofen and metoprolol). Comparison of these data with previous human data challenges ability of the rat SPIP model for predicting differences in human regional intestinal permeability of low-to-medium permeability drugs. The effect of PEs on drug permeability in the colon was similar to previously reported data from the rat jejunum. In contrast, their effect on the transport of Cr-EDTA—a clinical marker for mucosal barrier integrity—was significantly higher in the colon than in jejunum. These results indicate that the risk of using PE for increasing colonic drug permeability is higher than the potential medical reward.

## Figures and Tables

**Table 1:** Some physicochemical properties and Biopharmaceutics Classification System (BCS) classification of the four model drugs [14].

Compounds (BCS Class)	MM (g/mol)	pK_a_	PSA	HBA/HBD	Log P	Log D_7.4_	Log D_6.5_
Atenolol (III)	266	9.6 ^b^	88.1	4/4	0.18	−2.0	<−2.0
Enalaprilat (III)	348	3.17 ^b^/7.84 ^a^	102.1	6/3	−0.13	−1.0	−1.0
Metoprolol (I)	267	9.6 ^b^	57.8	4/2	2.07	0.0	−0.5
Ketoprofen (II)	254	3.89 ^a^	54.2	3/1	3.37	0.1	0.8

^a^ acid, ^b^ base, HBA/HBD—hydrogen bond acceptor/donor, Log D_7.4/6.5_—n-octanol−water partition coefficient at pH 7.4/6.5, Log P—n-octanol−water coefficient, MM—molar mass, pKa—dissociation constant, PSA—polar surface area.

**Table 2:** The mean ±SD rat permeability (P_eff_) values for the four model compounds determined in the jejunum and colon at pH 6.5 and 7.4 (*n* = 6).

Conditions	Plasma Appearance P_eff_ (×10^–4^ cm/s)			
	Atenolol	Enalaprilat	Ketoprofen	Metoprolol
Jejunum pH 6.5	0.022 ± 0.01	0.005 ± 0.004	1.5 ± 1.1	0.28 ± 0.24
Jejunum pH 7.4	0.016 ± 0.005	0.004 ± 0.001	0.64 ± 0.15	0.17 ± 0.095
Colon pH 6.5	0.015 ± 0.007	0.009 ± 0.007	1.1 ± 0.3	0.41 ± 0.19
Colon pH 7.4	0.011 ± 0.005	0.006 ± 0.004	0.73 ± 0.14	0.38 ± 0.15

## Abbreviations

AME—absorption-modifying excipient, CL_Cr-EDTA_—clearance of ^51^Cr-EDTA, P_eff_—intestinal effective permeability, PE—permeation enhancer, SDS—sodium dodecyl sulfate, SPIP—single-pass intestinal perfusion
